# Thiol–Ene Photopolymerization: Scaling Law and Analytical Formulas for Conversion Based on Kinetic Rate and Thiol–Ene Molar Ratio

**DOI:** 10.3390/polym11101640

**Published:** 2019-10-10

**Authors:** Kuo-Ti Chen, Da-Chuan Cheng, Jui-Teng Lin, Hsia-Wei Liu

**Affiliations:** 1 Graduate Institute of Applied Science and Engineering, Fu Jen Catholic University, New Taipei City 24205, Taiwan; tony022199@msn.com; 2 Department of Biomedical Imaging and Radiological Science, China Medical University, Taizhong 400, Taiwan; dccheng@mail.cmu.edu.tw; 3 New Vision, Inc., New Taipei City 242, Taiwan; jtlin55@gmail.com; 4 Department of Life Science, Fu Jen Catholic University, New Taipei City 24205, Taiwan

**Keywords:** thiol–ene functional group, kinetic model, photopolymerization, crosslinking, curing depth

## Abstract

Kinetics and analytical formulas for radical-mediated thiol–ene photopolymerization were developed in this paper. The conversion efficacy of thiol–ene systems was studied for various propagation to chain transfer kinetic rate-ratio (R_K_), and thiol–ene concentration molar-ratio (R_C_). Numerical data were analyzed using analytical formulas and compared with the experimental data. We demonstrated that our model for a thiol–acrylate system with homopolymerization effects, and for a thiol–norbornene system with viscosity effects, fit much better with the measured data than a previous model excluding these effects. The general features for the roles of R_K_ and R_C_ on the conversion efficacy of thiol (C_T_) and ene (C_V_) are: (i) for R_K_ = 1, C_V_ and C_T_ have the same temporal profiles, but have a reversed dependence on R_C_; (ii) for R_K_ >> 1, C_T_ are almost independent of R_C_; (iii) for R_K_ << 1, C_V_ and C_T_ have the same profiles and both are decreasing functions of the homopolymerization effects defined by k_CV_; (iv) viscosity does not affect the efficacy in the case of R_K_ >> 1, but reduces the efficacy of C_V_ for other values of R_K_. For a fixed light dose, higher light intensity has a higher transient efficacy but a lower steady-state conversion, resulting from a bimolecular termination. In contrast, in type II unimolecular termination, the conversion is mainly governed by the light dose rather than its intensity. For optically thick polymers, the light intensity increases with time due to photoinitiator depletion, and thus the assumption of constant photoinitiator concentration (as in most previous models) suffers an error of 5% to 20% (underestimated) of the crosslink depth and the efficacy. Scaling law for the overall reaction order, defined by [A]*^m^*[B]*^n^* and governed by the types of ene and the rate ratio is discussed herein. The dual ratio (R_K_ and R_C_) for various binary functional groups (thiol–vinyl, thiol–acrylate, and thiol–norbornene) may be tailored to minimize side effects for maximal monomer conversion or tunable degree of crosslinking.

## 1. Introduction

Photopolymerization and crosslinking have been utilized in various medical and industrial applications [1,2,3,4,5]. Compared to thermal-initiated polymerization, photo-initiated polymerization provides the advantages of fast and controllable reaction rates and spatial and temporal control over the formation of the material without the need for high temperatures or harsh conditions [1,2]. Tissue engineering using scaffold-based procedures for chemical modification of polymers has been reported to improve their mechanical properties by crosslinking or polymerization with UV or visible light to produce gels or high-molecular-weight polymers [3]. Industrial applications to date include the development of materials for thin films, 3D bio-printing, and microfabrication [5,6,7], in which the kinetics and mechanisms of photopolymerization have been extensively studied theoretically and experimentally [7,8,9,10,11,12,13,14,15,16,17,18].

The UV curing system is roughly categorized into three units: the radical system, cationic system, and anionic system. The radical system is mainly used for conventional UV curing systems. Thiol–ene photopolymerization (TEP) is based on the radical catalyzed addition of a thiol to a vinyl functional group, in which cross-linked polymer networks are formed via a two-step growth mechanism: (i) propagation of a thiyl radical through a vinyl functional group, (ii) followed by chain transfer from the resulting carbon radical to a thiol functional group, regenerating the thiyl radical [18,19,20,21,22]. In comparison, thiol–Michael photopolymerization (TMP) involves anion-mediated addition of a multifunctional thiol to an electron-deficient vinyl group of a multivinyl component [23,24,25,26,27]. Both TEP and TMP exhibit the advantages of typical photopolymerizations, including rapid reaction, optical clarity, negligible oxygen inhibition, solvent tolerance, high reaction yields, excellent mechanical properties, and they do not require solvents for processing [21,22,23]. In addition, TMP exhibits delayed gelation, and minimal byproduct formation and homopropagation effect of the vinyl group. Depending on the specific ene selected, TEP generally proceeds via a step-mode or mixed-mode chain-growth radical mechanism, and exhibits reaction kinetics strongly dependent on the electronic density of the ene (electron-rich vs. electron-poor) as well as the thiol and ene structures [18,19]. Both computational and experimental investigations have been performed to evaluate the role of solvents, monomers, and catalysts on this reaction mechanism [18,19,20,21,22,23,24,25,26,27].

The mechanism and kinetics of TEP utilizing a tetrafunctional thiol monomer copolymerized with acrylate, norbornene, vinyl ether, and vinyl-silazane-functionalized ene monomers were modeled and experimentally characterized by Cramers et al. [18] and Reddy et al. [20]. They demonstrated that reaction orders in TEP systems are controlled by the ratio of thiyl radical propagation (k_P_) to chain transfer (k_CT_) kinetic parameters, R_K_ = k_P_/k_CT_, which is also related to the electron density of the vinyl group and the carbon radical stability, which could provide spatiotemporal control over the course of the reaction. The scaling law for the functional group concentration of thiol, [A], and ene, [B], given by [A]*^m^*[B]*^n^*, with *m* and *n* ranging from 0 to 2, is related to the type of ene and the rate ratio. For example, for R_K_ >> 1, polymerization rates are first order in ene concentration (or *n* = 1.0) and nearly independent of the thiol concentration (or *m* = 0); in contrast, *m* = 1.0 and *n* = 0 for R_K_ << 1. For R_K_ values near unity, polymerization rates are approximately 0.5 order in both the thiol and ene functional group concentrations (*m* = *n* = 0.5). However, a scaling law of *m* = 0.4 and *n* = 0.6 has been found in an acrylate system (with R_K_ = 13) due to contributions from homopolymerization [18].

In general, competitive propagation/chain transfer process must occur, with one of the elementary reaction steps often becoming rate-limiting. These processes also determine the rate-limiting step and time to reach the gel-point conversion [24]. The temporal profiles of thiol and ene functional groups were successfully modeled and compared with measured data by Cramers et al. [18] with various rate-ratio R_K_ and concentration-ratio R_C_, except for the thiol–norbornene system which has a strong viscosity effect. Therefore, their model excluding the viscosity effect predicted a higher efficacy than their measured data. Although Cramers et al. [18] presented the temporal profiles of the functional groups based on numerical solutions, however, no analytical formulas were reported. Most of the previous models [18,19,20,21,22,23,24,25,26,27] have been based on oversimplified assumptions of constant photoinitiator (PI) or photosensitizer (PS) concentration (without depletion), and thus the light intensity in these models follows the conventional Beer–Lambert law (BLL), which is only valid for optically thin polymers and will suffer huge errors in optically thick polymers [15,16].

This study will focus, for the first time, on the analytical formulas for the conversion efficacy for various kinetic rate ratios and functional group concentration molar ratios. A set of more general kinetic rate equations was derived, including viscosity and homopolymerization effects. Numerical data were analyzed using our analytic formulas and compared with the experimental data of Cramers et al. [18]. We also demonstrated that our model for a thiol–acrylate system including homopolymerization effects, and for a thiol–norbornene system including viscosity effects, fit much better with the measured data than the model of Cramers et al. [18]. Furthermore, the dynamic light intensity resulted by the PI depletion has been discussed, in which the optically thin assumption in previous models [18,19,20,21,22,23,24,25,26,27] caused errors of 5% to 20% on crosslink depth and efficacy. We have also discussed the scaling law for the overall reaction order, defined by [A]^m^[B]^n^, and governed by the types of ene and the rate ratios. This study provides useful guidance for free radical photopolymerization via the roles of the dual ratios (R_K_ and R_C_) in various binary functional groups, including thiol–vinyl, thiol–acrylate, and thiol–norbornene.

## 2. Materials and Methods

### 2.1. Photochemical Kinetics

**Figure 1:**
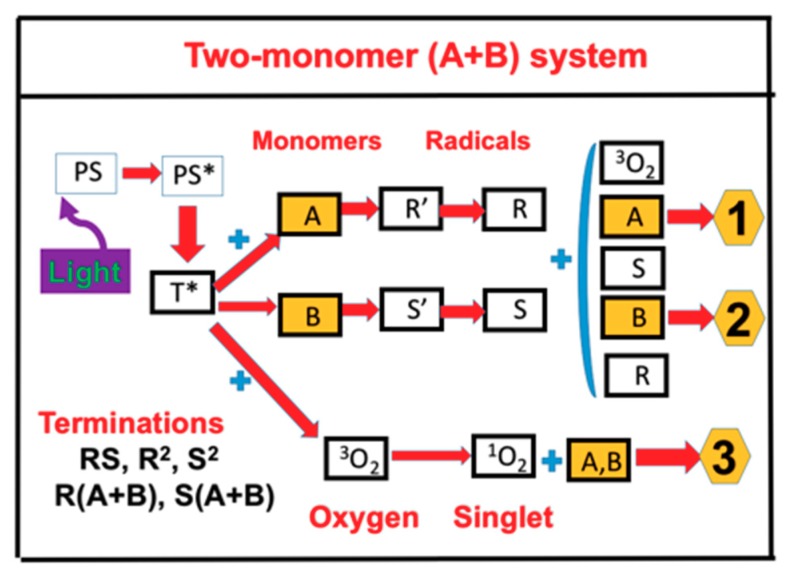
Schematics of three photochemical pathways in a two-monomer system, A and B, in the presence of ground state oxygen ^3^O_2_, for radical-mediated pathways (1 and 2), and oxygen-mediated pathway (3). PS is the ground state photosensitizer (PS), having excited and triplet states PS* and T*; terminations include unimolecular, bimolecular recombination, and inter-radical coupling [15,17].

As shown in Figure 1, a two-monomer system [A] and [B] involves three crosslinking pathways: two radical-mediated (or electron transfer) pathways (1 and 2), and one oxygen-mediated (or energy transfer) pathway (3). The ground state photosensitizer (PI) is excited to its triplet excited state T* by a quantum yield (*q*). In a type I process, T* interacts directly with [A] and [B] to form the intermediate radicals R’ and S’, which then produce the reactive R and S which could interact with oxygen ^3^O_2_, [A], [B], or bimolecular terminations R^2^ and S^2^. For a type II (or oxygen-mediated) process, T* interacts with ^3^O_2_ to form a singlet oxygen ^1^O_2_, which could interact with [A], [B], or be relaxed to ^3^O_2_. Both type I and type II reactions can occur simultaneously in photopolymerization, and the ratio between these processes depends on the type of photosensitizer (PS) or photoinitiator (PI) used, the concentrations of PS or PI, substrate monomers and oxygen, the kinetic rates involved in the process, and the light intensity, dose, PI depletion rate, etc. Greater details of these kinetics were published in our earlier work [13,14,15]. In a thiol–ene polymerization system, the functional groups are insensitive to oxygen inhibition; therefore, it can be treated as a type-I-dominant system.

**Figure 2:**
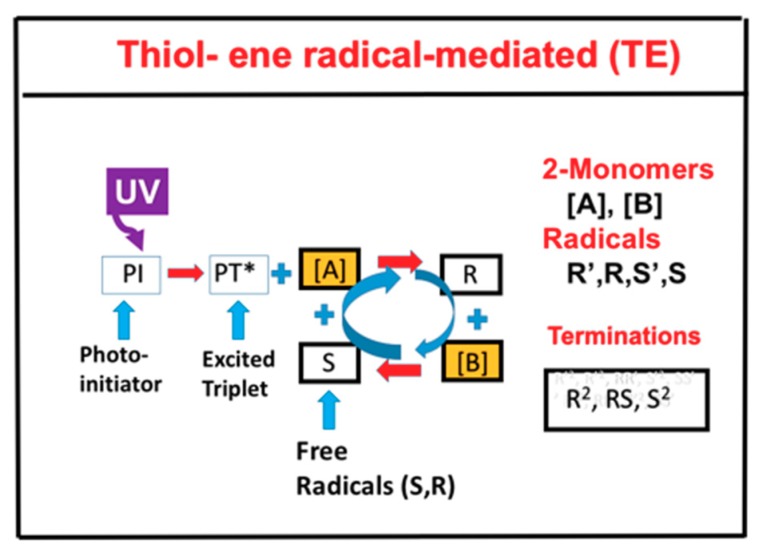
Schematics of UV-light initiated photopolymerization kinetics of thiol [A] and ene [B] functional groups, in which the thiyl radical R reacts with [B] to form a carbon radical (S), which reacts with the thiol and regenerates R to form the reaction cycle; R and S could interact with each other or be terminated by bimolecular recombinations, S^2^ and R^2^.

For a thiol–ene system, as shown in Figure 2, the photochemical pathways are more simple and can be shown by two-step growth mechanism, which involves (i) propagation of a thiyl radical (R) through an ene functional group [B] to form a carbon radical (S), and (ii) chain transfer from the resulting carbon radical (S) to a thiol functional group [A], regenerating the thiyl radical (R) which reacts with [A] to form the reaction cycle, where R and S could be coupled and also terminated by bimolecular recombination, or react with [A] and [B] in general.

The thiol–ene pathways of Figure 2 are a special situation of the more general pathways of Figure 1, where the functional groups are insensitive to oxygen inhibition and can be treated as a type-I-dominant system, neglecting oxygen-mediated type II reactions. In addition, thiol monomer, [B], do not couple directly with thiyl radical (R), but with the carbon radical (S) to regenerate R to form the reaction cycle. Therefore, the rate equations for thiol–ene system (Figure 2) are the reduced form of the two-monomer system (Figure 1). For simplicity, the intermediate radicals in Figure 2 (R’ and S’) are not shown in the kinetic scheme, but they have been included in our kinetic equations and calculations.

Using the following short-hand notation: C for the concentration of PI ground state (with an initial value C_0_); [A] and [B] for the concentrations of the thiol and ene functional groups; [R’] and [S’] for the intermediate radicals, and [R] and [S] for the reactive radicals, we obtained a set of general rate equations associated with the two-monomer system shown by Figure 2, as follows [13,17].

(1) $$\frac{\partial C}{\partial t}\;=-\mathrm{bIg}(\left[A\right]+\left[B\right]+{\mathrm{kg}}^{\prime }C\left[[O_{2}\right])C+R_{E}$$

(2) $$\frac{\mathrm{dI}\left(z,t\right)}{\mathrm{dz}}\;=-A\left(z,t\right)I\left(z,t\right)$$

(3) $$\frac{\partial \left[R\right]}{\partial t}\;=2k_{T1}{\left[R^{\prime }\right]}^{2}-(2k_{T1}{\left[R\right]}^{2}+{\;k}_{12}\left[R\right]\left[S\right]+k^{\prime \prime }\left[R\right][O_{2}])-G$$

(4) $$\frac{\partial \left[S\right]}{\partial t}\;=\;2k_{T2}{\left[S^{\prime }\right]}^{2}-(2k_{T2}\left[S{]}^{2}+{\;k}_{12}\left[R\right]\left[S\right]+k^{\prime \prime }\left[S\right][O_{2}\right])+G$$

(5) $$\frac{\partial [O_{2}]}{\partial t}=-\left[\mathrm{bIg}C+k^{\prime \prime }\left(\left[R\right]+\left[S\right]\right)][O_{2}\right]+P$$

(6) $$\frac{\partial \left[A\right]}{\partial t}\;=\;-\;R_{T}\;\left[A\right]$$

(7) $$\frac{\partial \left[B\right]}{\partial t}\;=-\;R_{T}\;\left[B\right]$$

(8) $$R_{T}=\;{\mathrm{bICgg}}^{\prime }[O_{2}]+k^{\prime }\left(\left[R\right]+\left[S\right]\right)$$

(9) $$G=k_{41}\left[R\right]\left[A\right]-k_{42}\left[S\right]\left[B\right]$$

(10) $$A\left(z,t\right)=2.3\left[\left(\;a^{\prime }-b^{\prime }\right)C+{b^{\prime }C}_{0}+Q\right]$$

where *b* = *aq*I(z,t); *q* is the quantum yield of the PI excited state; *a* = 83.6*wa’*; *w* is the wavelength of light (in cm); and I(z,t) is the light intensity with a unit of mW/cm^2^. *a’* and *b’* are the molar extinction coefficients (in 1/mM/%) of the initiator and the photolysis product, respectively; and *Q* is the absorption coefficient of the monomer and the polymer repeat unit.

R_E_ is the regeneration of PI ground state given by R_E_ = (k_91_[R] + k_92_[S])[O_2_] + 2k_T1_R^2^ + 2k_T2_S^2^; *g* = 1/(k_57_ + k_37_[O_2_] + [A] + [B]), *g’* = 1/(k_68_+ k_48_C + [A] + [B]), *k* = (k_11_/k_8_); k_37_ = k_3_/k_7_, k_57_ = k_5_/k_7_; k_68_ = k_6_/k_8_, k_48_ = k_4_/k_8_, where k_5_ and k_3_ are the relaxation rate of the PI triplet state (T*) and the coupling rate of T* and oxygen; k_71_ and k_72_ are the coupling rate of T* and monomers [A] and [B]; k_88_, k_81_, and k_82_ are the coupling rates of singlet oxygen and monomers; k_41_, k_42_, k_51_, and k_52_ are the coupling of radicals R and S and monomers. Greater detail and derivation of the above equations may be found in References [13,15], which also show the kinetic equations for the triplet state (T*) and singlet oxygen [X]. Therefore, Equation (1) to (10) of this article describe the quasi-steady-state form of Equation (1) from Reference [13] under the quasi-steady-state condition dT*/dt = d[X]/dt = 0 = d[R’]/dt = d[S’]/dt = 0, which also defines the steady-state value of radicals [R’] and [S’]. Substituting these values into Equations (3) and (4), we obtain the steady-state reactive radicals [R] and [S]:

(11) $$2k_{T1}{\left[R\right]}^{2}+\;k\left[R\right][O_{2}]+G-\mathrm{bICg}\left[A\right]=0$$

(12) $$2k_{T2}\left[S{]}^{2}+k^{\prime }\left[S\right][O_{2}\right]-G\;-\mathrm{bICg}\left[B\right]=0$$

For a thiol–ene system, which has the advantages of being relatively uninhibited by oxygen. Therefore, we may ignore the oxygen related terms k” [R][O_2_] and k” [S][O_2_], in Equations (1) to (10). Without oxygen inhibition, k_57_ << [A]+[B], g([A]+[B]) = 1 for k_72_ = k_71_, and g’[O_2_] = 0. We further assumed, as proposed by Cramer et al. [10], that the consumption rates of the thiol and ene functional groups could be set as equal, d[A]/dt = d[B]/dt, which allowed us to solve for Equations (6) and (7) for [S], thus solving Equation (11) for [R]. Substituting these steady-state values, [S] and [R], into Equation (8), we obtained the simplified rate equations of monomers [A] and [B] as follows.

(13) $$\frac{d\left[A\right]}{\mathrm{dt}}\;=-R_{p}$$

(14) $$\frac{d\left[B\right]}{\mathrm{dt}}=-R_{p}\left(1+H_{M}\right)$$

(15) $$R_{p}=K\sqrt{0.5bIC/G}$$

(16) $$G={\left(Fk_{P}\left[B\right]\right)}^{-2}+{\left(k_{CT}\left[A\right]\right)}^{-2}+{\left(Fk_{P}k_{CT}\left[B\right]\left[A\right]\right)}^{-1}$$

(17) $$H_{M}=(k_{CV}/\;k_{CT})(\left[B\right]/\left(D\left[A\right]\right)$$

where H_M_ is the revised term for the homopolymerization effect, with *D* = 1 + k_CT_[B]/(Fk_P_[A]). *K* = k_P_/k_T_^0.5^ is an effective rate constant; k_52_ = k_CT_, k_T1_ = k_T2_ = k_T_, are, respectively, the rate constants for chain transfer, propagation, and termination; and k_52_ = k_CV_ is the rate of the homopolymerization effect, H_M_, which is given by the k_52_S[B] term of Equation (9); however, we ignored the k_51_S[A] term, since [A] (thiol) has weaker homopolymerization than [B] (ene).

In Equation (16), we also revised the propagation rate constant k_P_, by a reduction factor (F) for the viscosity effect on the ene, given by F = 1 − d’C_EFF_, where d’ is a fit reduction rate and C_EFF_ is the conversion efficacy of the ene group given by C_EFF_ = 1 − exp(−S), where S is given by the time integral of [A]/[A]_0_, or the solution of Equation (13). The viscosity effect reduces the available free volume or decreases k_P_ in the diffusion-controlled region [11]. Without the extra two consumption terms of the ene group viscosity and homopolymerization effects, H_M_ = 0 (or k_CV_ = 0) and F = 1, Equations (13) and (14) have the same total reaction rate function, R_P_, which is symmetric with regards to [A] and [B] such that their conversion efficacies are also symmetrically related. These features are further demonstrated later in the paper by our numerical data.

The above revised kinetic equations can be reduced to those of Cramer et al. [18] under the following three assumptions: (i) homopolymerization and viscosity effects are neglected (with H_M_ = 0 and F = 1); (ii) the PI concentration is a constant, or Equation (1) dC/dt = 0 (or bIt << 1, for small doses), and (iii) light intensity is a constant, or Equation (2), dI/dt = dI/dz = 0, which is valid only for a short exposure time, or an optically thin polymer.

Accurate solutions of Equations (13) to (17) require numerical simulations. For analytical formulas, we used approximated analytic formulas for the light intensity and the PI concentration, such that we did not need to solve for Equation (1), and the expressive closed forms of I(z,t) and C(z,t) allowed us to solve analytically for the first-order solutions of Equations (13) and (14) for the chain and propagation limited cases.

### 2.2. Analytical Formulas for Efficacy

The monomer conversion efficacy for a bimolecular termination process is given by C_EFF_ = 1 − [M]/[M]_0_ = 1 − exp(−S) with [M] = [A] or [B], and the S function is given by the solution of Equations (13) and (14) as follows. Analytical solutions are available for the two limiting cases defined by the ratio of chain (k_P_) and propagation rate (k_CT_), defined as R_K_ = k_P_ /k_CT_: in Case i, k_P_ << k_CT_ (chain limited), and in Case ii, k_P_ >> k_CT_ (propagation limited).

For Case i, k_P_[B] << k_TC_[A], Equation (15) becomes

(18) $$R_{p}=K\sqrt{0.5bIC}\;Fk_{p}\left[B\right]$$

Using the above R_P_ and solving for the first-order solution of Equation (14), for the case of F = 1 (viscosity effect is neglected) and F’ = 0 (or homopolymerization effect is neglected), we obtained the first-order solution of [A] and [B], which allowed us to calculate F’ and the conversion efficacy of the ene, C_V_ = 1 − [B]/[B]_0_,

(19) $$C_{V}=\;1-\exp\left(-S\right)$$

(20) $$S=\overset{t}{\underset{0}{\int }}KFk_{p}\left(1+H_{M}\right)\sqrt{0.5bIC}\mathrm{dt}$$

which also gives [B] = [B]_0_exp(−S), and solve for Equation (13); thus, we obtained the conversion efficacy of the thiol, defined by C_T_ = 1 − [A]/[A]_0_,

(21) $$C_{T}=\;1-\;\left({\left[B\right]}_{0}/{\left[A\right]}_{0}\right)S_{T}$$

(22) $$S_{T}=\overset{t}{\underset{0}{\int }}KFk_{p}\sqrt{0.5bIC}\exp\left(-S\right)\mathrm{dt}$$

For Case ii, k_P_[B] >> k_TC_[A], Equation (15) becomes

(23) $$R_{p}=K\sqrt{0.5bIC}\;k_{CT}\left[A\right]$$

Similarly, solving for Equations (13) and (14), we obtained the second-order conversion efficacy for thiol (C_T_) and ene (C_V_), as follows.

(24) $$C_{T}=\;1-\exp\left(-S^{\prime }\right)$$

(25) $$S^{\prime }=\overset{t}{\underset{0}{\int }}Kk_{CT}\sqrt{0.5bIC}\mathrm{dt}$$

(26) $$C_{V}=\;1-\left({\left[A\right]}_{0}/{\left[B\right]}_{0}\right)S_{V}$$

(27) $$S_{V}=\overset{t}{\underset{0}{\int }}Kk_{CT}\left(1+H_{M}\right)\sqrt{0.5bIC}\;\exp\left(-S^{\prime }\right)\mathrm{dt}$$

### 2.3. The Second-Order Solution

Equations (19) to (27) are the first-order solutions for the limiting cases of k_P_[B] >> k_TC_[A] and k_P_[B] << k_TC_[A]. To include the high-order terms for the case of comparable k_P_ and k_CT_ with k_P_[B] > k_TC_[A], Equation (22) was revised to (1 − d) S, with d = (k_P_/k_CT_) [B’]/[A’], which is a decreasing function of the first-order solution product [B’]/[A’]. Therefore, the conversion efficacy of [B] is an increasing function of the concentration ratio [A]_0_/[B]_0_. Similarly, for k_P_[B] < k_TC_[A], Equation (25) was revised to (k_CT_/k_P_) (1 − 1/d) S’, which is a decreasing function of [A’]/[B’]. Therefore, the conversion efficacy of [A] is a decreasing function of [A]_0_/[B]_0_. This feature of the conversion efficacy of [A] and [B] can be seen by the symmetrical formulas, as shown by Equations (21) and (26). This feature is consistent with the measured data shown in Figure 3 of Cramer et al. [18] for a thiol–vinyl ether system with k_P_ = 1.2k_CT_. The common feature is that for k_P_ = k_CT_, the conversions of [A] and [B] have symmetrical formats and are proportional to the ratios [A]_0_/[B]_0_ and [B]_0_/[A]_0_, respectively. This symmetry is broken when k_P_ and k_CT_ are not equal, or when there are other consumption factors of [B], such as viscosity or homopolymerization effects. Greater details are discussed later.

### 2.4. Effects of Viscosity and Homopolymerization

To include the effects of homopolymerization given by H_M_, the first-order solutions of [A] and [B] can be substituted into Equation (20) for the H_M_ function; we thus obtained the second-order conversion efficacy defined by a revised S-function of Equation (20), which resulted a higher efficacy for [B] and [A], as predicted by Equation (20), for the case of k_P_[B] << k_TC_[A]. In comparison, when k_P_[B] >> k_TC_[A], H_M_ does not affect the first-order solution of [A], as shown by Equation (25), but it reduces the efficacy of [B], as shown by Equation (27). We confirmed these features by numerical solution, as detailed later in the paper.

To include the viscosity effects, or when F < 1 in Equation (16), the free volume was reduced when crosslink efficacy increased. The reduction factor (F) only affected the propagation rate constant Fk_P_ in Equation (17), but not that in Equation (23). Therefore, it only reduced the efficacy of [B]. We propose F = 1 − [1 − exp(−S’)], with S’ proportional to the efficacy and given by a fit function S’ = 2[1 − exp(−mb’I_0_t)]/(mb’I_0_)^0.5^, where m and b are fit parameters to be found later by comparing with the measured data. The reduction factor is proportional to the available free volume, or a decreasing function of the conversion efficacy, which is proportional to S’. To include both the homopolymerization and viscosity effects, F and F’ are needed to solve for Equation (3). Numerical solutions, shown later, demonstrated the above features predicted by our analytical formulas.

### 2.5. Dynamic Light Intensity

Solving Equations (1) and (2) for the light intensity, I(z,t), and PI concentration, C(z,t), concentration, we numerically found S and S’ and then the conversion functions, C_V_ and C_T_. We further derived the analytical form of conversion efficacy, which required closed forms of I(z,t) and C(z,t), as follows. Using our previously developed approximated analytical formulas [13,15]

(28) $$I\left(z,t\right)=I_{0}\exp\left[-A^{\prime }z\right]$$

(29) $$C^{\prime }\left(z,t\right)=C_{0}\exp\left[-Bt\right]$$

(30) $$A^{\prime }\left(z,t\right)=2.3\left(a^{\prime }C_{0}+Q\right)-B^{\prime }t$$

where B = bI_0_exp(−A”z), B’ = 2.3(a’ − b’)C_0_I_0_bz, with A” as the averaged absorption given by A” = 1.15(a’ + b’) C_0_ + 2.3Q. Note that the –B’t term represents the decrease of A’, or increase of light intensity due to PI depletion. Equation (29) is the approximated solution of Equation (1) with oxygen ignored, or k_88_[X] = 0 and g[A] + g’[B] = 1. Using Equations (28) and (29), we obtained the analytical form of Equation (20) for the cases of F = 1 and F’ = 0:

(31) $$S=K\sqrt{0.5bXI_{0}C_{0}}\;E\;(z,t)$$

(32) $$E\left(z,t\right)=\left[1-\exp\left(-B^{\prime \prime }t\right)\right]/B^{\prime \prime }$$

where B” = 0.5(B − B’) and X = exp [−2.3(a’C_0_ + Q) z]. Equation (31) gives the close form of Equation (20) for C_V_, and also allowed us to numerically integrate Equation (6) to obtain C_T_ for Case i. Similarly, for Case ii and Equation (25), S’ = (k_CT_/k_P_) S, which gave a close form of C_V_, and C_T_ from Equation (27). Note that Equation (30) defines the dynamic feature of the light intensity, which is an increasing function of time due to the depletion of the PI concentration. It also provides the nonlinear depth (z) dependence of A’z, given by B’. The above analytical formulas provide useful information with which to analyze and predict the critical roles of each of the influencing factors without numerically solving the coupled equations.

### 2.6. Gelation Time

The critical (gelation point) conversion, C_CT_, may be defined by the classical Flory−Stockmayer equation [10]:

(33) $$C_{CT}=1/\sqrt{R_{C}\left(f_{T}-1\right)\left(f_{V}-1\right)}$$

where f_T_ and f_V_ are the functionalities of thiol and ene monomers, respectively; and R_C_ = [A]_0_/[B]_0_ is the thiol–ene stoichiometric initial molar ratio. For the case of neglected induction time, we found that the gelation time can be given by solving the exposure time of Equations (31) and (6) under the critical condition of Equation (33). For example, for the case of k_P_[B] << k_TC_[A], we solved for Equations (31) and (33) to obtain the gelation time (T_GEL_) for the ene:

(34) $$T_{GEL}=\left(1/B^{\prime \prime }\right)\ln\left[1/\left(1-B_{1}\right)\right]$$

(35) $$B_{1}=B^{\prime \prime }L/\left(K\sqrt{0.5b}\right)$$

(36) $$L=\ln\left[1/\left(1-C_{CT}\right)\right]$$

Similarly, we obtained the gelation time for the case of k_P_[B] >> k_TC_[A] given by Equation (20), given by the same formula as Equation (34), but Equation (35) was revised to:

(37) $$B_{1}=B^{\prime \prime }L/[k_{TC}K\sqrt{0.5b}]$$

However, there are no analytic formulas for C_T_ for the case of k_P_[B] << k_TC_[A], or C_V_ for the case of k_P_[B] >> k_TC_[A] due to the complex integrations of Equations (20) and (25).

### 2.7. Crosslink Depth

A crosslink depth (Z_C_) is defined by when the conversion efficacy is higher than a critical value, C_T_ > C_CT_, or when S > S_CT_ with S_CT_ = ln [1/(1 − C_CT_)]. Using Equation (31), and with S = S_CT_ = 2 (or C_CT_ = 0.86), Z_C_ is related to the crosslink time (T_C_) by:

(38) $$T_{C}=\left(\frac{1}{B^{\prime \prime }}\right)\ln\left[2B^{\prime \prime }/\left(K\sqrt{0.5bXI_{0}C_{0}}\;\right)-1\right]$$

Note that Equation (38) is a nonlinear function of Z_C_; therefore, there is no analytical formula for Z_C_ vs. T_C_. However, this formula can be found by plotting the curve of T_C_ vs. Z_C_, then rotating the axis to show the curve of Z_C_ vs. T_C_. Numerical results based on Equation (38) are shown later in the paper.

## 3. Results and Discussion

### 3.1. Efficacy Spatial Profiles

**Figure 3:**
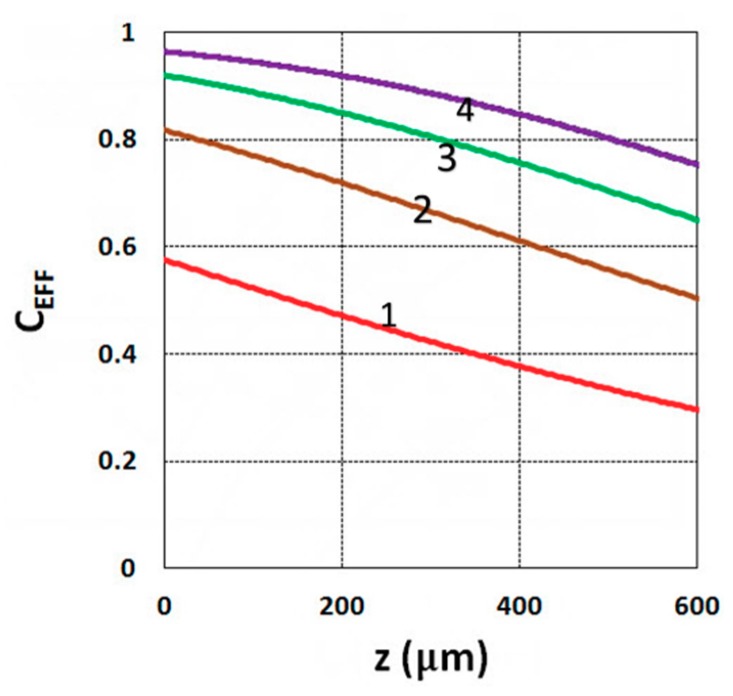
Conversion efficacy vs. depth (z) for t = (10, 20, 30, 40) s, given by curves 1, 2, 3, and 4, respectively, for a fixed light intensity I_0_ = 3 mW/cm^2^, C_0_ = 0.1%, b = 0.001, a’ = 130 (1/cm/mol), K = 5.0, b’ = Q = 0, B’ = 0.

**Figure 4:**
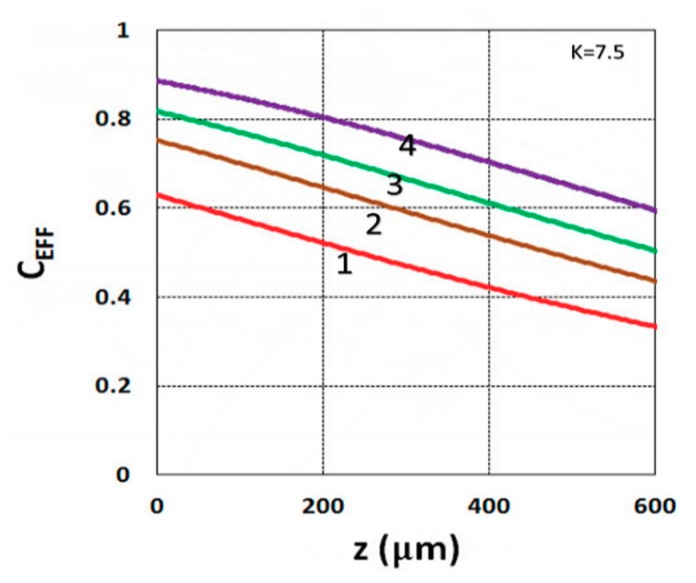
Conversion efficacy vs. depth (z) for I_0_ = (1, 2, 3, 5) mW/cm^2^, given by curves 1, 2, 3, and 4, respectively, for t = 20 s.

As shown by Equation (31), the S function also defines the monomer conversion efficacy given by C_EFF_ = 1 − exp(−S), and S has a transient state function E(z,t), governed by the dynamic profiles of the light intensity I(z,t) and PI concentration, C(z,t), given by Equations (28) and (29). Based on Equations (19) and (31), the spatial profiles of the conversion efficacies of the monomer [B] (or ene) are shown in Figure 3; for a fixed light intensity, efficacy is an increasing function of exposure time (t), but a decreasing function of the depth (z). Similarly, Figure 4 shows that the efficacy is an increasing function of light intensity (for a fixed exposure time). However, for very large exposure times, with an S function approaching its steady state, with E(z,t) = 1 in Equation (32), the efficacy becomes an increasing function of z, as reported by our previous studies [15,16] which also discussed the scaling laws of the S function in greater details.

### 3.2. Dynamic Profiles of the Light Intensity

**Figure 5:**
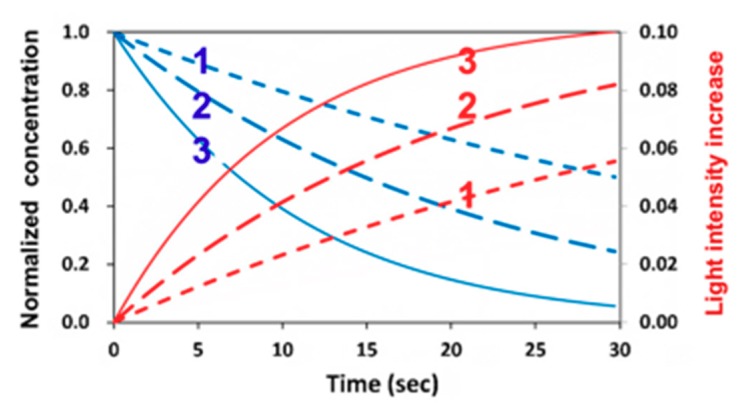
Dynamic profiles of normalized photoinitiator (PI) concentration (blue curves) and light intensity increases dI (red curves) for b = (0.05, 0.1, 0.2), for curves 1,2,3.

**Figure 6:**
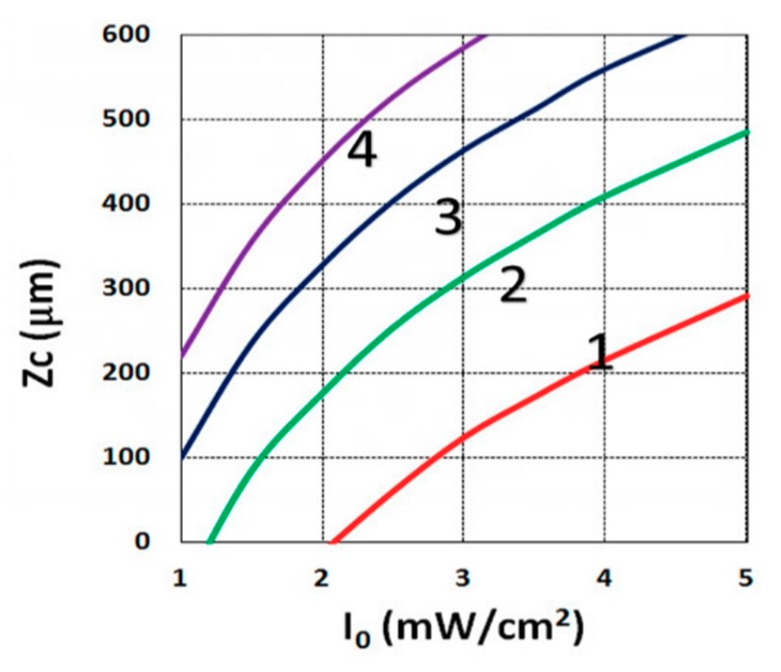
Crosslink depth (Z_C_) vs. light intensity (I_0_) for t = (15, 20, 25, 30) s, given by curves 1, 2, 3, and 4, respectively, for C_0_ = 0.1%, based on Equation (14), for B’ = 0.

Figure 5 shows the dynamic profiles of the PI-normalized concentration and the increase of light intensity obtained by the numerical solution of Equation (1). Depending on the coupling parameters b and A, as shown by Equation (30), the depletion of C(z,t) causes the increasing I(z,t), defined by an increasing percentage dI = [I(z,t) − I_0_]/I_0_, which is also a decreasing function of the depth (z), per the Beer–Lambert law. Figure 6 shows an increase of 6% to 10% light intensity (at z = 100 µm) for b = 0.05 to 0.2, a’ = 200 (1/%/mM), b’ = 100 (1/cm/%), Q = 50 (1/cm), and I_0_ = 5 mW/cm^2^. Figure 6 shows that larger b values had a faster PI depletion and hence a larger light intensity increase. For thick polymers (>100 µm), the conversion efficacies of the thiol (C_T_) and ene (C_V_) functional groups are also depth (z)-dependent, as well as time-dependent. The example shown in Figure 5 demonstrates that the assumption of constant light intensity and PI concentration is valid only for optically thin (<100 µm) polymers under a small dose (<1.0 mJ/cm^2^), i.e., under the condition of Equation (9c) with B’ = 2.3(a’ − b’) bC_0_I_0_tz < 0.1. For optically thick polymers under a larger dose, with bI_0_tz = 0.1 to 0.2, the thin-polymer model assumption will cause an error of 5% to 20%, depending on the depth of light propagation or polymer thickness, and its absorption coefficient. The influence of B’ on the crosslink depth and efficacy is shown later in the paper. Note also that, as shown by Equation (3), the photoinitiation rate function (R_P_) is proportional to the product of I(z,t) and C(z,t), which are two competing parameters, as shown by Figure 5. Therefore, there are optimal values of I(z,t) and C(z,t), as shown earlier in Figure 3 and Figure 4.

### 3.3. Crosslink Depth Profiles

**Figure 7:**
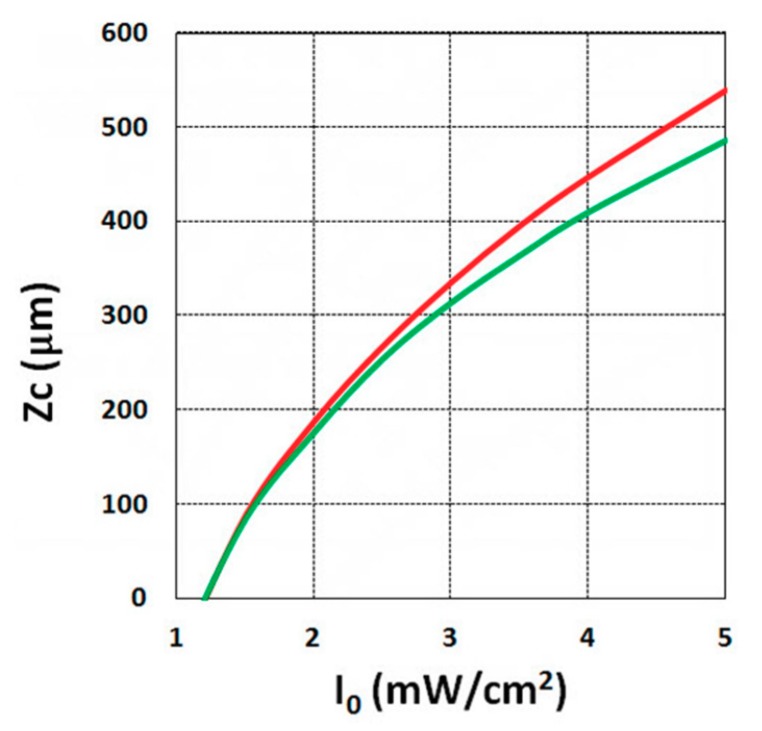
Crosslink depth (Z_C_) vs. light intensity (I_0_), for B’ = 0 (green curve), and B’ = 0.06I_0_z > 0 (red curve) for C_0_ = 0.1%, t = 20 s.

**Figure 8:**
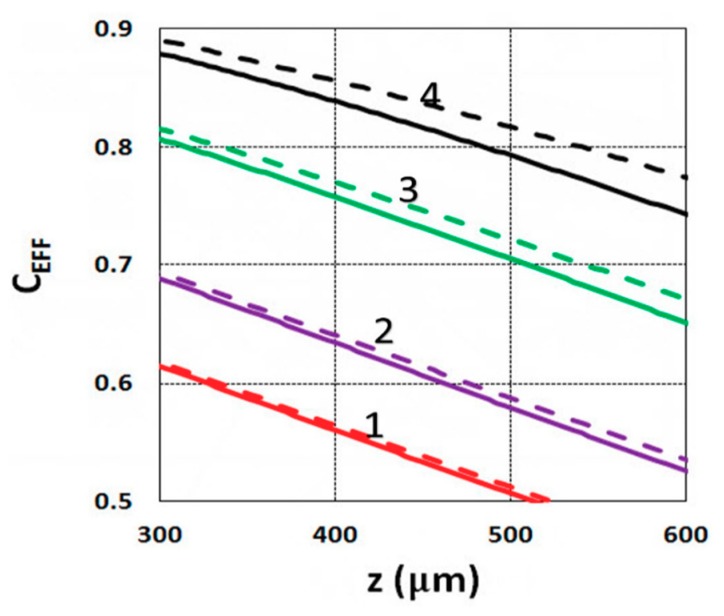
Efficacy vs. depth (z) based on Equation (14) for I_0_ = (1, 2, 3, 5) mW/cm^2^, given by curves 1, 2, 3, and 4, respectively, at t = 20 s, C_0_ = 0.1%; for B’ = 0 (solid curves), and B’ = 0.06I_0_z (dashed curves).

Using the analytical formula Equation (38), we were able to investigate the roles of PI, oxygen concentration, light intensity, and exposure time in crosslink depth. Figure 6 shows that crosslink depth (Z_C_) (for the case of B’ = 0) is an increasing function of light intensity and exposure time. Figure 7 shows the influence of dynamic light intensity due to PI depletion given by B’ in Equation (30): that the assumption of B’ = 0 suffers an error of 10% to 20% (underestimated) of Z_C_ for depths of 300 to 500 µm. Therefore, the assumption is valid only for optically thin polymer thinner than 200 µm for the case of absorption constant a’ = 130, and thinner than 100 µm for a stronger absorption of 260 (1/cm/%), or when C_0_ = 0.2%, as shown by Equation (9), B’ = 2.3(a’ − b’) C_0_I_0_bz. The influence of B’ on the efficacy profiles is shown in Figure 8, namely that neglecting the B’ factor also results in an underestimation of the efficacy.

### 3.4. Numerical Results of Conversion

This section shows the numerical results of the conversion efficacy of thiol and various ene functional groups by solving Equation (1) to (10). These numerical data were analyzed by our analytic formulas and compared with the experimental data of Cramers et al. [18]. We also demonstrated that our modeling for a thiol–acrylate system including homopolymerization effects, and for a thiol–norbornene system including viscosity effects, fit much better with the measured data than the model of Cramers et al. [18] for the high efficacy region.

**Figure 9:**
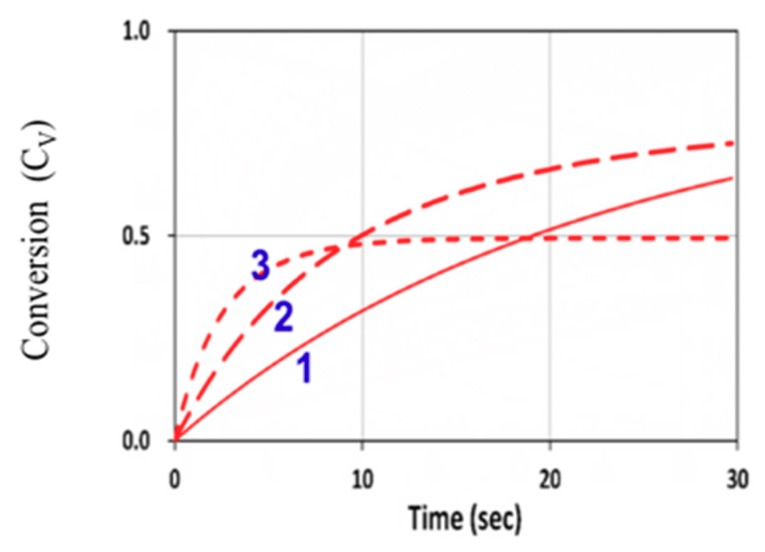
Conversion efficacy of ene (C_V_) vs. time at various light intensity of I_0_ = (1, 5, 25) mW/cm^2^, given by curves 1, 2, and 3, respectively, for b = 0.05, kp = 0.1 × 10^5^, k_CT_ = 2 × 10^5^ (with a rate ratio R_K_ = kp/k_CT_ = 0.2), k_T_ = 1 × 10^6^ (L/mol.s), and [A]_0_ = [B]_0_ = 5.0 mol/L, neglecting the viscosity and homopolymerization effects (or k_CV_ = 0 and F = 1).

Figure 9 shows the conversion efficacy of ene, [B], for a fixed z = 100 µm, with a rate ratio R_K_ = k_P_/k_CT_ = 0.2, for various light intensity of I_0_ = (1, 5, 25) mW/cm^2^, which demonstrates that, for a fixed light dose, higher light intensity has a higher transient efficacy, but a lower steady-state conversion. These numerically obtained features were also predicted by Equation (10), and may be compared with Figure 5.

**Figure 10:**
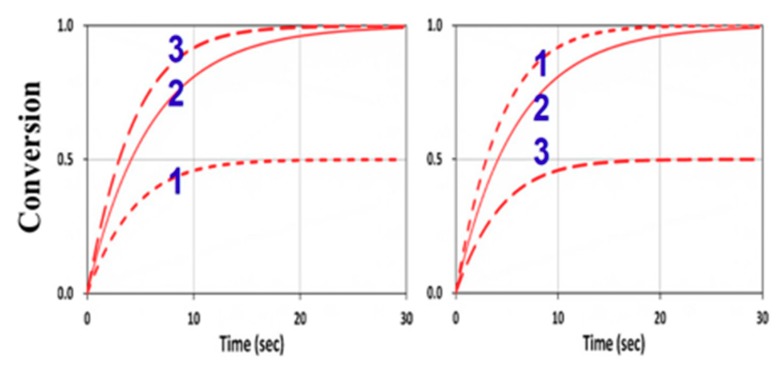
Conversion efficacy of ene (left) and thiol (right) for R_K_ = 1.0 and various concentration ratios R_C_ = [A]_0_/[B]_0_ = (0.5, 1.0, 2.0), given by curves 1, 2, and 3, respectively, neglecting the viscosity and homopolymerization effects (k_CV_ = 0 and F = 1).

We next explored the roles of the rate ratio R_K_ = k_P_/k_CT_ for various concentration ratios R_C_ = [A]_0_/[B]_0_. For k_CV_ = 0 and F = 1, the G function of Equation (16) was the same for Equations (13) and (14), which are symmetric equations. Therefore, [A] and [B] had identical solutions, except that their dependence on R_C_ is reversed, i.e., [B] was governed by [B]_0_/[A]_0_ and [A] by [A]_0_/[B]_0_. These opposite dependences on the concentration ratio R_C_ lead to the reversed curves of 1 and 3 in Figure 10 for [B] and [A]. These features seem unexpected numerically, but they were clearly predicted by our analytical formulas, Equations (24) and (26), for the case that k_CV_ = 0 and F = 1, or when the homopolymerization and viscosity effects were neglected.

### 3.5. Analysis of Measured Data

**Figure 11:**
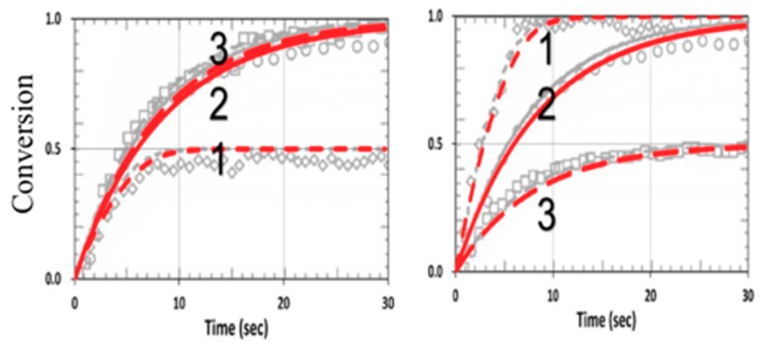
Calculated conversion efficacy of vinyl (left) and thiol (right), for k_P_ = k_CP_ = 1.0 × 10^5^, (or R_K_ = 1.0), kT = 1 × 10^6^ (L/mol.s), and various concentration ratios R_C_ = [A]_0_/[B]_0_ = (0.5, 1.0, 2.0), given by curves 1, 2, and 3, respectively; the background gray circles are the measured data of Cramer et al. [18].

Figure 11 shows the conversion in a thiol–vinyl system, using the same parameters used in Figure 4 from Cramers et al. [18], with R_K_ = k_P_/k_CT_ = 0.2 and k_CV_ = 0, for various R_C_ = (0.5., 1.0, 2.0). Our calculated curves fit very well with the measured data of Cramers et al. (with fit b = 0.01).

**Figure 12:**
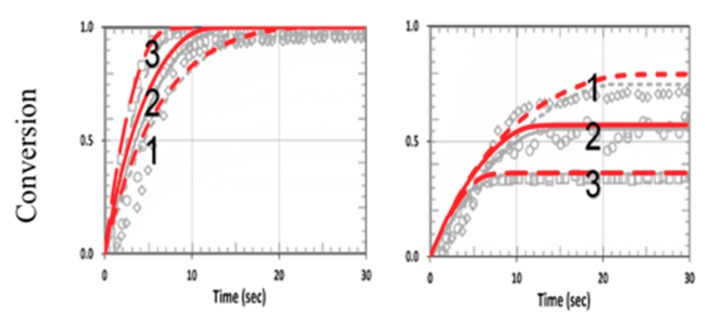
Calculated conversion efficacy of acrylate (left) and thiol (right), for k_P_ = 2.2 × 10^5^, k_CP_ = 0.17 × 10^5^ (or R_K_ = 13), and various concentration ratios R_C_ = [A]_0_/[B]_0_ = (0.5, 1.0, 2.0), including homopolymerization effects (with k_CV_ = 0.25 × 10^5^). The background gray circles are the measured data of Cramer et al. [18].

Figure 12 shows the effects of homopolymerization (with k_CV_ = 0.25 × 10^5^) in a thiol–acrylate system, using the same parameters used in Figure 13 from Cramers et al. [18] with kp/k_CT_ = 13, but also including the dynamic light intensity and PI depletion. The modeling data of Cramers et al. were consistent with ours for specific values of b and z. However, for different PI concentration profiles (or different b), or polymer thicknesses (z), the conversion efficacies of thiol and ene were changed accordingly. As predicted by Equations (24) and (25), the conversion efficacy of thiol [A] did not affected by H_M_ or k_CV_. In contrast, as predicted by Equation (27), the efficacy of acrylate [B] increased when k_CV_ increased. This feature was also shown by our numerical data presented in Figure 11, in which our model fit very well the measured data of Cramer et al. [18], except for the transient region in which we neglected the induction time (or lag period) due to the initial interaction of the functional groups and the impurities or oxygen. Therefore, a further improved model including induction time would be needed for strong induction systems as discussed by Claudino et al. [22].

**Figure 14:**
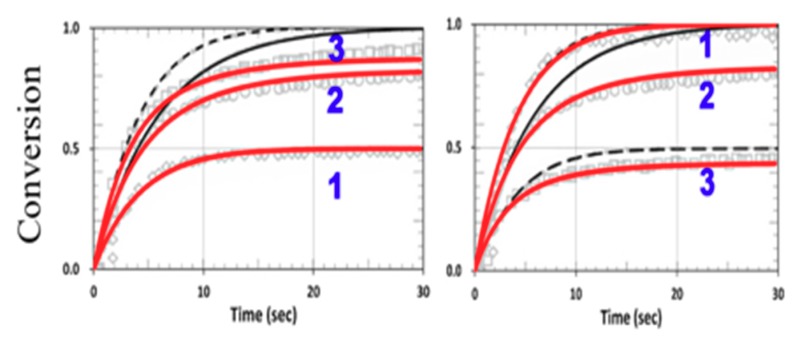
Calculated conversion efficacy of norbornene (left) and thiol (right), including the viscosity effects (with a reduction factor, F < 1), for various concentration ratios R_C_ = [A]_0_/[B]_0_ = (0.5, 1.0, 2.0), for curves 1,2,3; with k_P_ = k_CT_ = 1.0 × 10^5^ (R_K_ = 1.0). The calculated curves without (in black dash) and with (in red) the viscosity effects are also shown.

Figure 14 shows the viscosity effect on the conversion efficacy using the same parameters shown in Figure 2 from Cramers et al. [18], with k_P_ = k_CP_ = 1.0 × 10^5^ (or R_K_ = 1.0) for a thiol–norbornene system. Note that the model-predicted conversions of Cramers et al. (shown by black curves) were higher than their measured data, due to their assumption of a constant reaction rate (k_P_). In our revised model, we included a reduction factor (F < 1) in Equation (22) for the viscosity effect, which reduces the available free volume specifically for the high efficacy region. We propose that F = 1 − [1− exp(−S’)], with S’ proportional to the efficacy and given by a fit function S’ = 2[1 − exp(−mb’I_0_t)]/(mb’I_0_)^0.5^, with m = 0.5 and b’ = 0.03 fit to the measured data, as in Figure 2 of Cramers et al. [18]. The reduction factor is proportional to the available free volume, or a decreasing function of the conversion efficacy, which is proportional to S’. As shown in Figure 14, a much better fit to the measured curves was found in our revised model (shown by red curves). Note that without the viscosity effect (or k_CV_ = 0, F = 1), Figure 14 reduces to Figure 11, in which the symmetrical feature of C_V_ and C_T_ becomes asymmetrical, resulting from the viscosity effect, which mainly affects the propagation parameter of norbornene (k_P_). This reduction factor (F) affects both C_V_ and C_T_ specifically for the case of k_P_ = k_CT_ (or R_K_ = 1.0). Note that the modeling curves in Figure 2 of Cramers et al. [18] are the same as in our Figure 11 (with k_CV_ = 0, F = 1).

### 3.6. General Features of Conversion Efficacy

Our numerical results for the conversion efficacies C_T_ (for thiol [A]) and C_V_ (for ene [B]) showed that the roles of the reaction rate ratio, R_K_ = k_P_/k_CT_, and the concentration ratio, R_C_ = [A]_0_/[B]_0_, were consistent with our predicted results based on analytical formulas which provided more general features for the roles of R_K_ and R_C_, summarized as follows:

Without viscosity (with F = 1) or homopolymerization (or k_CV_ = 0) effects, [A] and [B] have an equal overall polymerization rate (R_P_); C_V_ (C_T_) is an increasing (decreasing) function of the ratio R_C_. For R_K_ = 1 (or kp = k_CT_), C_V_ and C_T_ have the same temporal profiles, but have a reversed dependence on R_C_, as shown by Figure 10.

For R_K_ << 1, [B] and C_V_ are almost independent from R_C_, with a second-order correction proportional to R_C_ having asymmetrical dependence on R_C_, given by (1 − d), with d = R_K_/R_C_, as shown by Figure 11.

For R_K_ >> 1, [A] and C_T_ are almost independent from R_C_, but the second-order correction is inversely proportional to R_C_, an opposite trend to that of C_V_. As predicted by Equation (25), the first-order solutions (with neglected d = 0) of C_T_ and C_V_ were independent from R_C_.

For R_K_ << 1 and with the homopolymerization effect considered (with k_CV_ > 0, F’ > 0), a revised S function of Equation (21) and Equation (27) predicts that C_V_ and C_T_ would have the same profiles and both be decreasing functions of k_CV_. In comparison, for the case of R_K_ >> 1, k_CV_ does not affect [A], as shown by Equation (26), which predicts that C_V_ is an increasing function of k_CV_, but C_T_ is a slightly decreasing function of k_CV_, due to its second-order correction.

With the presence of viscosity effect, or when F < 1, in Equation (22), the free-volume is reduced when crosslink efficacy increases. The reduction factor (F) only affects the propagation rate constant Fk_p_ in Equations (20) and (22), but not in Equation (25). Therefore, the viscosity effect does not affect the efficacy for the case of R_K_ >> 1, and affects the efficacy for other ratios of k_P_ and k_CT_, where the viscosity effect reduces the efficacy of [B], as predicted by Equation (22).

As predicted by the analytical formula of Equation (31) and the numerical data of Figure 9, for a fixed light dose, higher light intensity has a higher transient efficacy, but a lower steady-state conversion. This unique and unusual feature of the light intensity dependence is the result of a bimolecular termination. In contrast, in type II unimolecular termination, the conversion is mainly governed by the light dose, rather than its intensity.

For optically thick polymers, the influence of dynamic light intensity due to PI depletion is given by B’ in Equation (30), which predicts that the assumption of B’ = 0 (as in most previous models) suffers an error of 5% to 20% (underestimated) for a crosslink depth (Z_C_) ranging 300 to 500 µm, and also underestimates the efficacy, as shown by Figure 7 and Figure 8.

Scaling laws for the functional group concentration of thiol, [A], and ene, [B] are given by [A]*^m^*[B]*^n^.* For R_K_ >> 1, the polymerization rates are first order in the ene concentration (or *n* = 1.0) and nearly independent of the thiol concentration (or *m* = 0); in contrast, *m* = 1.0 and *n* = 0 for R_K_ << 1. For R_K_ values near unity, polymerization rates are approximately 0.5 order in both thiol and ene functional group concentrations (*m* = *n* = 0.5). However, a scaling law of *m* = 0.4 and *n* = 0.6 was found in an acrylate system (with R_K_ = 13), due to contributions from homopolymerization.

Based on the above-described general features for thiol–ene polymer systems, it is possible to tailor the two ratios, R_K_ and R_C_, and choose the appropriate ene functional group to minimize side effects such as viscosity and homopolymerization for maximal monomer conversion or tunable degree of crosslinking. The binary thiol–vinyl system used in this study may be expended for multiple-component systems, such as the ternary thiol–ene–ene and thiol–ene–acrylate systems reported by Reddy et al. [20]. Furthermore, the model and formulas developed for the free-radical-mediated thiol–ene system may be extended (to be published elsewhere) for anionic chain process such as base-catalyzed, thiol-Michael addition reactions [26]. Further information on the monomer properties discussed in this article and the factors influencing photopolymerization kinetics and optimal materials with low molecular weight, low viscosity, and in situ polymerization mechanisms have been reported in earlier publications [28,29,30].

## 4. Conclusions

We demonstrated that our model for a thiol–acrylate system including homopolymerization effects, and for a thiol–norbornene system including viscosity effects, fit much better with the measured data than the previous model. Furthermore, we found that the dynamic light intensity due to photoinitiator depletion cannot be neglected in optically thick polymers. The efficacies of the thiols and enes depend on both R_K_ and R_C_, which may be tailored together with the choice of ene functional group to minimize side effects such as viscosity and homopolymerization for maximal conversion efficacy.

## Figures and Tables

**Figure 13:**
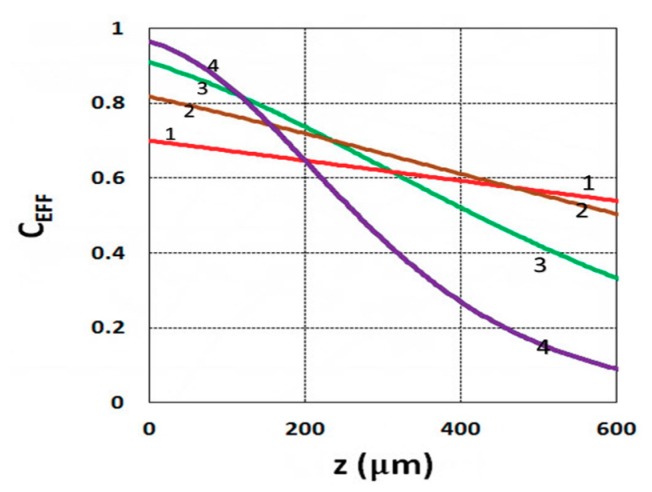
Conversion efficacy vs. depth (z) for C_0_ = (0.05, 0.1, 0.2, 0.4) %, given by curves 1, 2, 3, and 4, respectively, for I_0_ = 3 mW/cm^2^ at t = 20 s.
